# Management of Atrial Fibrillation in Elderly Patients: A Whole New Ballgame?

**DOI:** 10.3390/jcm14072328

**Published:** 2025-03-28

**Authors:** Iris Parrini, Fabiana Lucà, Carmelo Massimiliano Rao, Roberto Ceravolo, Sandro Gelsomino, Carlo Ammendolea, Laura Pezzi, Nadia Ingianni, Donatella Del Sindaco, Adriano Murrone, Giovanna Geraci, Claudio Bilato, Giuseppe Armentaro, Angela Sciacqua, Carmine Riccio, Furio Colivicchi, Massimo Grimaldi, Fabrizio Oliva, Michele Massimo Gulizia

**Affiliations:** 1 Department of Cardiology, Mauriziano Hospital, 10128 Turin, Italy; irisparrini@libero.it; 2 Department of Cardiology, Grande Ospedale Metropolitano (GOM) of Reggio Calabria, Bianchi Melacrino Morelli Hospital, 89129 Reggio Calabria, Italy; 3 Department of Cardiology, Santa Maria degli Ungheresi Hospital, Polistena, 89024 Reggio Calabria, Italy; massimo.rao@libero.it; 4 Department of Cardiology, San Giovanni Paolo II Hospital, 88046 Lamezia Terme, Italy; roberto_ceravolo@yahoo.it; 5 Cardiovascular Department, Maastricht University, 6229HX Maastricht, The Netherlands; sandro.gelsomino@gmail.com; 6 Department of Cardiology, San Martino Hospital, 32100 Belluno, Italy; carlo.ammendolea@ulss.belluno.it; 7 Department of Cardiology, Ospedale Civile dello Spirito Santo, 65100 Pescara, Italy; 8 Cardiology, ASP Trapani, Marsala District, 91022 Castelvetrano, Italy; nadiaing@hotmail.it; 9 Cardiology Unit, Nuovo Regina Margherita Hospital, 00100 Rome, Italy; donatelladelsindaco@virgilio.it; 10 Cardiology Department, Città di Castello Hospital, 06012 Citta di Castello, Italy; adriano.murrone@gmail.com; 11 Cardiology Department, Sant’Antonio Abate Hospital ASP Trapani, 91100 Erice, Italy; giovannageraci@hotmail.com; 12 Department of Cardiology, Vicenza Ovest Hospital, Arzignano, 36100 Vicenza, Italy; claudio.bilato@aulss8.veneto.it; 13 Department of Internal Medicine and UO of Geriatrics, Magna Graecia University of Catanzaro, 88100 Catanzaro, Italy; giuseppearmentaro91@gmail.com (G.A.); sciacqua@unicz.it (A.S.); 14 Cardio-Vascular Department, AORN Sant’Anna e San Sebastiano, 81100 Caserta, Italy; carminericcio8@gmail.com; 15 Department of Emergency and Acceptance, Presidio Ospedaliero San Filippo Neri, ASL Roma 1, 00135 Rome, Italy; furio.colivicchi@gmail.com; 16 Department of Cardiology, F. Miulli Hospital, Acquaviva delle Fonti, 70021 Bari, Italy; m.grimaldi@miulli.it; 17 Department of Cardiology, Niguarda Hospital, 20162 Milano, Italy; fabri.oliva@gmail.com; 18 Department of Cardiology, Garibaldi Nesima Hospital, 95122 Catania, Italy; michele.gulizia60@gmail.com

**Keywords:** atrial fibrillation (AF), rate control, rhythm control, elderly patients, heart failure (HF), cognitive decline, anticoagulation

## Abstract

Atrial fibrillation (AF) is the most prevalent sustained supraventricular arrhythmia, particularly in older adults, with its incidence increasing dramatically with age. This condition is a significant contributor to morbidity and mortality, being closely linked to an elevated risk of heart failure, ischemic stroke, systemic thromboembolism, and dementia. The complexities of managing AF in the elderly arise from age-related physiological changes, comorbidities, frailty, and the challenges of polypharmacy. Therapeutic strategies must balance efficacy and safety, tailoring interventions to the individual’s health status, life expectancy, and personal preferences. This review explores the latest evidence-based approaches to managing AF in elderly patients, focusing on the nuanced application of rate and rhythm control strategies, anticoagulation, and emerging insights into the relationship between AF and cognitive impairment.

## 1. Introduction

Atrial fibrillation (AF) represents the most common sustained cardiac arrhythmia worldwide, with an increasing prevalence that is projected to double by 2050, primarily driven by global population ageing [1]. While AF can occur in any age group, its prevalence increases exponentially with age, affecting up to 10% of individuals over 80 years [1,2].

The clinical significance of AF lies in its strong association with debilitating complications, including HF, systemic embolism, and cognitive dysfunction, which collectively impose a substantial burden on healthcare systems and significantly impair QoL. In the elderly, AF management is particularly challenging due to the confluence of age-related pathophysiological changes, such as atrial fibrosis and structural remodelling, along with the presence of multiple comorbidities, frailty, and polypharmacy [3]. Cognitive decline and functional limitations may also hinder treatment adherence and complicate decision-making [4]. These challenges necessitate a personalized multidisciplinary approach that integrates clinical guidelines [5,6], taking into consideration each patient’s individual needs [3].

This review aims to provide a comprehensive overview of the optimal therapeutic strategies for managing AF in elderly patients, emphasizing the importance of individualized care. We will explore the role of rate and rhythm control, the evolving landscape of anticoagulation therapy, and the potential implications of AF for cognitive health. By synthesizing current evidence and clinical recommendations, this review seeks to inform best practices and highlight areas for future research.

## 2. Materials and Methods

### 2.1. Search Strategy

The systematic review was conducted in compliance with the Preferred Reporting Items for Systematic Reviews and Meta-Analyses (PRISMA) guidelines [7] and the Cochrane Handbook [8]. A comprehensive literature search was executed across PubMed, Embase, and Cochrane databases without any language or time restrictions. PubMed was designated as the primary database. The search strategy employed the following terms: (((“atrial fibrillation”) AND (“management of treatment”)) AND (“rate control” OR “rhythm control”)) AND (“elderly” OR “older”). Two authors (F.L. and I.P.) developed the search strategy, which was subsequently reviewed and approved by a third author (C.M.R.). The actual search was conducted by C.M.R., while two independent reviewers evaluated the eligibility of the articles and assessed the risk of bias. The risk of bias at the individual study level was assessed using the Risk of Bias in Non-Randomized Studies of Interventions (ROBINS-I) tool. Two reviewers conducted independent evaluations of the included studies. In the event of disagreement, a third reviewer (G.G.) was consulted to reach a consensus. The evaluation criteria included the following: (1) confounding factors, (2) participant selection, (3) classification of interventions, (4) deviations from intended interventions, (5) missing data, (6) outcome measurement, (7) selection of reported outcomes, and (8) overall bias. The assessment followed the guidelines provided in the Cochrane Handbook. Risk-of-Bias Visualization (robvis) software v0.3.0 was utilized to generate the ROBINS-I bias assessment plot.

### 2.2. Selection Process

The selection of articles adhered to predefined inclusion criteria, namely (1) studies involving human subjects, (2) full-text articles focused on atrial fibrillation management, (3) studies providing sufficient data on atrial fibrillation presence, and (4) studies with a minimum of 10 participants. Exclusion criteria included the following: (1) non-human studies, (2) case reports, (3) previous reviews or meta-analyses, (4) editorials, (5) studies lacking specific data, (6) studies reporting unrelated outcomes, and (7) studies without relevant data on the targeted aspect.

### 2.3. Results

**Figure 1:**
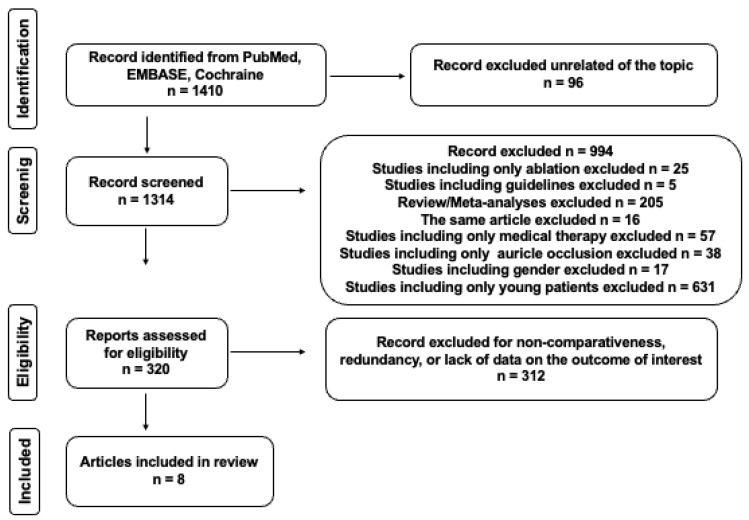
PRISMA diagram on the selection of included studies.

The initial search yielded 1410 articles published over a five-year period. After applying the inclusion and exclusion criteria, 1090 articles were excluded due to irrelevance. Subsequently, 320 articles underwent screening based on their titles and abstracts. Two hundred and fifty-three articles were selected for further evaluation (see the flowchart in Figure 1). Ultimately, eight articles met the eligibility criteria and were included in the final analysis. A summary of these studies is provided in the subsequent section. (See Table 1).

## 3. Pathophysiological Susceptibility to AF in the Elderly

### 3.1. Structural and Electrophysiological Alterations

**Figure 2:**
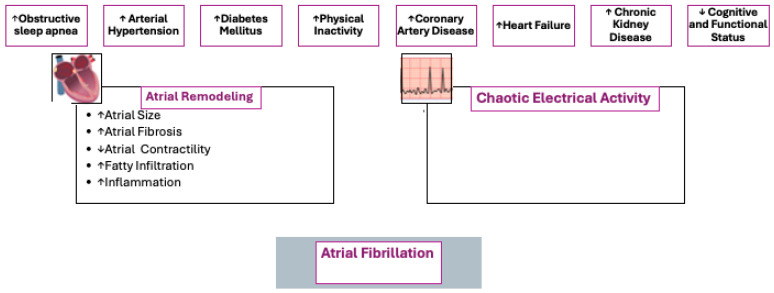
AF development in the elderly. This figure illustrates the multifactorial pathophysiology underlying AF development in the elderly. It highlights the key risk factors contributing to atrial remodelling and chaotic electrical activity, ultimately leading to the onset of AF. In the figure are illustrated several well-established risk factors for AF, including obstructive sleep apnea, AH, DM, physical inactivity, coronary artery disease, heart failure, chronic kidney disease, and cognitive and functional status. These factors are known to play a critical role in promoting structural and electrical changes in the atria. The pathological changes that occur within the atrial myocardium, such as increased atrial size, atrial fibrosis, impaired contractility, fatty infiltration, and inflammation are shown. These structural alterations create a substrate that is prone to abnormal electrical conduction and re-entry circuits. ↑: Increase; ↓: Reduction.

The heightened susceptibility of older adults to AF results from a combination of age-related structural and functional changes in the atrial myocardium [17]. Progressive atrial fibrosis—often accompanied by amyloid deposition—leads to stiffening of the atrial walls, reduced myocardial compliance, and loss of elasticity [18]. These alterations promote atrial dilation, thereby establishing a substrate conducive to the development and maintenance of AF (Figure 2) [19]. Moreover, ageing is associated with slowed electrical conduction, prolonged atrial refractory periods, and reduced sinus node activity, creating an electrophysiological environment favourable for arrhythmogenesis [20]. The cumulative impact of lifetime exposure to cardiovascular risk factors such as arterial hypertension (AH) [21], diabetes mellitus (DM), and obesity further increases the likelihood of AF onset [6].

### 3.2. Comprehensive Geriatric Assessment in AF Management

Given these complexities, evaluating elderly patients with AF requires a multidimensional approach [22,23]. A comprehensive geriatric assessment (CGA) is essential to guide therapeutic decisions by evaluating frailty, functional capacity, cognitive status, and nutritional health [24]. Tools such as the FRAIL scale [25], the 5 m gait speed test, the Mini Nutritional Assessment (MNA), and the Mini-Cog test [26,27,28,29] help identify patients who are most likely to benefit from tailored interventions while minimizing the risk of complications or iatrogenic harm. Additional assessments, including the five-item Geriatric Depression Scale and bioimpedance analysis for sarcopenia [28], further contribute to understanding patient resilience and treatment tolerance. Risk stratification models like Rockwood’s Frailty Index, which integrates functional status, cognitive performance, and comorbidities [25], have proven useful in predicting adverse outcomes and guiding clinical decisions.

Importantly, identifying frailty and advanced age should not be considered an absolute contraindication for procedural interventions; instead, these factors highlight the need for a thorough evaluation to optimize outcomes and reduce the risk of adverse events [24].

## 4. Integrated Care Pathways

Current guidelines advocate for an integrated patient-centred approach to AF management in elderly populations [6]. This strategy not only involves effective arrhythmia control but also emphasizes optimizing anticoagulation therapy to prevent thromboembolic events. Tailoring interventions according to each patient’s life expectancy, functional status, and overall health is paramount [30].

### 4.1. Tailored Interventions

Individualized care ensures that treatment decisions align with patient-specific goals and circumstances. For instance, adapting therapy based on overall health and comorbidities can significantly improve both clinical outcomes and quality of life [31].

### 4.2. Medication Management and Deprescribing

Studies such as STOPP/START [32] and STOPPFrail [33] highlight the importance of deprescribing inappropriate medications—particularly in end-of-life care—to prioritize symptom control over aggressive disease prevention. This careful adjustment of therapy helps mitigate the risks of polypharmacy and adverse drug events in elderly patients [31,34].

### 4.3. Addressing Gaps in Guideline Adherence

Despite advances in AF management, research like the STEEER-AF study [13] has revealed significant gaps in adherence to clinical guidelines across multiple European countries. These findings underscore the need to enhance both patient and physician education to ensure a consistent implementation of evidence-based practice [30].

## 5. Rhythm Control

### 5.1. Comparison of Rhythm and Rate Control

**Figure 3:**
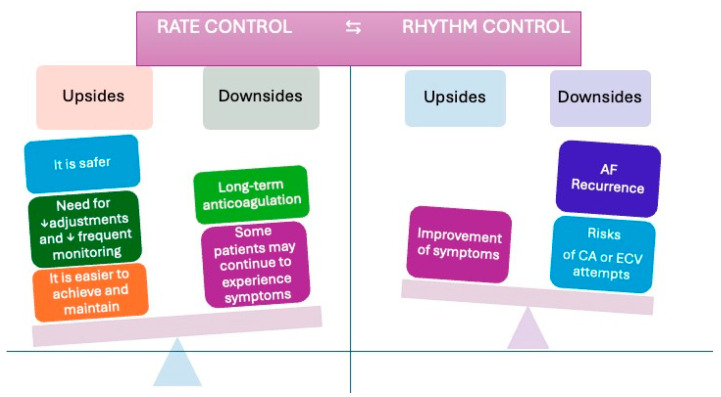
Advantages and disadvantages of rate control and rhythm control strategies in the elderly. This figure illustrates the comparative upsides and downsides of two management strategies for AF in elderly patients. Rate control is associated with a lower risk of adverse events, which is particularly beneficial for elderly patients with multiple comorbidities. Although adjustments and periodic monitoring are required, the extent is generally lower than rhythm control, which is advantageous for elderly individuals. This strategy is more straightforward to achieve and maintain over the long term, fitting the needs of elderly patients who may have limited mobility. Elderly patients often need continuous anticoagulation therapy to mitigate the risk of thromboembolism, which can increase the risk of bleeding. Some elderly patients may continue to experience symptoms despite adequate rate control, affecting their quality of life. The rhythm control strategy effectively alleviates symptoms associated with AF, thereby enhancing the quality of life for elderly patients who are symptomatic despite rate control. However, elderly patients are more prone to experiencing recurrent episodes of AF. The procedural risks and potential complications related to CA or ECV are heightened in elderly patients due to age-related frailty and comorbidities. In summary, while rate control offers a safer and more manageable approach with fewer monitoring requirements, it may not fully resolve symptoms. Conversely, rhythm control can improve symptoms but carries a higher risk of recurrence and procedural complications, which are more pronounced in the elderly population.

Several studies have demonstrated that both rhythm and rate control strategies effectively manage symptoms and preserve cardiac function in AF patients [35]. However, rhythm control appears to offer potential advantages in early-onset AF (defined as AF ≤ 1 year), although some controversy remains [36,37] (see Figure 3). Electrical cardioversion (ECV) remains the treatment of choice for hemodynamically unstable patients. In contrast, pharmacological cardioversion can be employed in stable patients with a short AF duration [38]. If the onset of AF is uncertain, ECV should be postponed until after at least four weeks of therapeutic anticoagulation with direct oral anticoagulants (DOACs) [38]. Conversely, when AF onset is clearly defined and occurs within 24 h, ECV may be performed immediately under appropriate anticoagulation [38].

### 5.2. Evidence from Clinical Studies

The EAST-AFNET 4 trial [36] evaluated rhythm control in patients over 75 years—or those over 65 with additional cardiovascular risk factors—with early-onset AF (≤1 year). Most participants received antiarrhythmic drugs, and 8% underwent catheter ablation (CA) as a first-line therapy. This study demonstrated a significant reduction in adverse cardiovascular outcomes in the rhythm control group compared to usual care, emphasizing the benefits of early intervention in selected patients.

These findings have been supported by additional evidence. In this sense, a study involving 2635 AF patients pointed out that early rhythm control reduced CV events in those with AF durations of less than one year, a benefit not observed in patients with longer AF durations [39]. An observational study with 20,611 patients aged ≥over 65 found that early rhythm control is safe, even in frail patients, further supporting the need for individualized therapeutic approaches [40]. Despite these positive results, specific guidelines for managing AF in frail elderly populations remain limited.

### 5.3. Antiarrhythmic Drug Therapy (AADs)

Rhythm control therapy also includes the use of antiarrhythmic drugs (AADs) [34,41]. Amiodarone has been shown to be effective in maintaining sinus rhythm but is associated with higher toxicity (pulmonary, thyroid, hepatic) and side effects such as *torsade de pointes* and bradycardia [42]. Despite these drawbacks, it may be considered a reasonable first-line option in patients with concomitant heart failure (HF) [6,43]. Dronedarone is similar to amiodarone but with a better safety profile. The ATHENA trial [44] showed that dronedarone reduces mortality and cardiovascular hospitalizations in patients with paroxysmal or persistent AF. However, it can cause QT interval prolongation and has comparable liver toxicity to other class III AADs [45]. In healthy patients without functional or cognitive limitations and with new-onset AF, flecainide has been shown to improve survival, addressing concerns related to class IC agents, which have been associated with increased mortality in ischemic heart disease [46]. Although sotalol is used in rhythm control, the survival benefit appears lower. A Cochrane systematic review noted increased mortality when sotalol was used to maintain sinus rhythm after cardioversion [46]. Moreover, in the context of polypharmacy, sotalol is generally not recommended for the elderly due to its risk of QT prolongation and fatal arrhythmias, leading to increased mortality [47]. It is also important to note that early-phase treatment with AADs may induce bradycardia, falls, or syncope, which might require pacemaker implantation [42,48].

The use of sotalol in polypharmacy is not recommended for the elderly because of the risk of QT prolongation and the occurrence of fatal arrhythmias with increased mortality [49].

### 5.4. Alternative Approaches for Refractory AF

For patients with refractory or severely symptomatic rapid AF who do not respond adequately to pharmacological therapies, an “ablate and pace” strategy can be considered in selected cases [50]. This approach involves the ablation of the atrioventricular (AV) node to achieve a complete heart block, followed by permanent pacemaker implantation to maintain an adequate ventricular rate. This strategy effectively alleviates symptoms and improves the quality of life in selected patients by interrupting the transmission of rapid atrial impulses to the ventricles. However, careful patient selection and a thorough assessment of comorbidities are essential to optimize outcomes and minimize procedural risks [5].

## 6. Rate Control

Rate control aims to relieve symptoms and prevent tachycardia-induced cardiomyopathy or HF [51].

It is generally preferred in patients with long-standing persistent AF, multiple comorbidities, polypharmacy, cognitive impairment, frailty, and a short life expectancy [42,51].

The RACE II study analyzed the optimal heart rate, showing no significant differences in mortality, hospital admissions for HF, arrhythmia-related symptoms, and thromboembolic complications when the heart rate was kept below 80 bpm per minute compared to 110 bpm. This study helped to highlight the importance of adequate heart rate control in patients with permanent AF [52].

Beta-blockers, diltiazem and verapamil, are the most used drugs for rate control in patients with AF and an ejection fraction (EF) above 40%. Beta-blockers and/or digoxin are preferred in patients with AF and an EF of 40% or less [6,53].

Combined therapy with multiple rate control drugs is recommended only when necessary to achieve the target heart rate. Careful follow-up is essential in elderly patients to avoid bradyarrhythmia, with particular attention paid to the use of AADs and their administration in sick sinus syndrome [48].

Among the AADs, digoxin has been shown not to reduce all-cause mortality but to decrease hospital admissions in HFrEF with a dose < 0.125 mg per day or lower in case of renal insufficiency [54]. Particular attention should be given to long-term discontinuation in HFrEF due to poorer outcomes after discontinuation [43].

In recent years, the therapeutic range for digoxin used in heart failure has been revised; the current recommended range is 0.5 to 0.9 ng/mL or less than 1.0 ng/mL. This range was defined based on the risk of toxicity of digoxin, which should not be used as a first-line drug in the treatment of AF because safer alternatives are available [55] (Figure 3).

## 7. Catheter Ablation in Elderly Patients

Catheter ablation (CA) has been considered an effective strategy in the elderly, particularly in those who remain symptomatic despite therapy.

In the CASTLE-HF study, ref. [56], which included HFrEF patients, transcatheter ablation was associated with improved survival and reduced morbidity, particularly in those with implantable cardiac devices. However, a recent meta-analysis [57] highlighted that in elderly patients (>75 years), transcatheter cardiac ablation is linked to lower safety, higher recurrence rates of AF, and an increased risk of procedure-related complications compared to younger individuals.

The CASTLE-HTx [58] study demonstrated that CA is particularly beneficial in patients with advanced HFrEF, especially in high-risk cases. Notably, a reduction in mortality was observed after three years of follow-up, underscoring the importance of long-term monitoring. Similarly, the AATAC-AF [59] trial, a multicenter randomized study involving 203 patients with persistent AF and HFrEF (NYHA class II–III, LVEF ≤ 40%), found that CA was superior to amiodarone in terms of rhythm control and left ventricular function improvement. However, the CA group exhibited higher mortality at the two-year mark, although AF recurrence was less frequent.

In frail patients, it may be prudent to prioritize rhythm control via pharmacological therapy rather than invasive interventions like CA, even when performed by experienced operators. Given the high recurrence rate of AF, ongoing monitoring and tailored therapeutic adjustments are essential, particularly in elderly patients, to optimize treatment adherence [31,60,61]. The CABANA study, which included 2204 patients with a mean age of 68, found no significant differences in mortality between CA and rate control strategies [62].

Ablate and pace therapy presents a viable alternative for elderly patients, mainly when drug therapy is ineffective or poorly tolerated. However, pacemaker-induced desynchrony may exacerbate left ventricular dysfunction, a limitation that can be mitigated through His bundle pacing. Biventricular pacing (BiVP) is another potential solution in patients with HFeEF [63]. The ablate and pace approach has been shown to reduce all-cause and CV mortality, rehospitalization, and stroke, with normal atrioventricular pacing offering more stable hemodynamics compared to the irregular pacing seen in AF [64,65].

Considering each patient’s specific risks and preferences, these findings underscore the need for a personalized treatment approach. This decision-making process should involve discussions with both the patient and their family, ensuring a comprehensive understanding of the treatment options and associated risks [31]. In high-volume centres, CA may still benefit elderly patients in good physical condition, particularly those with recent-onset AF, minimal comorbidities, small left atrial volume, and no significant fibrotic remodelling. However, in frail populations, CA carries higher risks, including periprocedural complications, longer hospital stays due to slower recovery, and increased AF recurrence [31].

Overall, CA is more effective than medical therapy in reducing AF recurrences and improving quality of life, as well as potentially lowering the risk of stroke [66]. Comorbidities remain a key predictor of major complications, highlighting the importance of individualized treatment strategies [67].

The decision to pursue CA in asymptomatic AF patients should be individualized, taking into account age, comorbidities, AF burden, and risk of disease progression. Current evidence suggests that asymptomatic patients have a higher likelihood of developing permanent AF, which may have long-term implications for stroke risk and HF development [68]. However, the absence of significant differences in primary clinical outcomes between symptomatic and asymptomatic patients raises questions about the benefit of early rhythm control in this population [68]. According to current guidelines, CA is primarily recommended for symptomatic patients to improve quality of life rather than to reduce mortality [66]. In elderly patients, the balance of risks and benefits must be carefully weighed: while ablation may offer rhythm control and prevent AF progression, procedural risks, including periprocedural complications and recurrence, are non-negligible [66]. Conservative management with optimal anticoagulation, rate control, and lifestyle modifications remains the first-line strategy in most asymptomatic elderly patients, particularly those with frailty or multiple comorbidities [67]. However, early CA may be a reasonable consideration in select cases—such as patients with high AF burden, early signs of left atrial remodelling, or a strong preference for rhythm control [69]. According to data from 36 studies including 217,850 participants, it has been pointed out that there are no significant differences between symptomatic and asymptomatic patients in terms of all-cause mortality, CV mortality, thromboembolic complications, stroke, hospitalization, or AMI. However, symptomatic individuals demonstrated a 33% higher likelihood of developing HF and a reduced risk of progressing to permanent AF. Additionally, symptomatic patients were more likely to receive antiarrhythmic drugs and undergo catheter ablation than those without symptoms [70].

These findings suggest that treatment decisions should be guided by individualized risk assessments rather than solely by symptom presence.

## 8. AF and Heart Failure

### 8.1. Prevalence and Bi-Directional Relationship

HF is a prevalent comorbidity among elderly patients with AF [71]. Approximately 50% of patients with AF develop HF [6], and those with AF have a five-fold increased risk of developing HF [72]. Longitudinal data from the Framingham study demonstrated that 57% of patients with new-onset HF had pre-existing AF, while 37% of those with new-onset AF developed HF [73]. These findings underscore the bidirectional relationship between AF and HF and the necessity of exploring strategies for prevention and management, particularly in elderly patients [74,75].

As recent guidelines recommend, the first step is to achieve euvolemia through diuretics to treat HF and achieve better heart rate control in AF [6].

### 8.2. Management Strategies

Guidelines emphasize the importance of achieving euvolemia using diuretics, which not only alleviates HF symptoms but also aids in achieving better heart rate control in AF [6]. For patients with HFrEF and concurrent AF, therapies such as beta-blockers, angiotensin receptor inhibitor neprilysin (ARNI), angiotensin-converting enzyme inhibitors (ACEis), and sodium glucose cotransporter type 2 inhibitors (SGLT2is) are recommended to improve prognosis irrespective of AF status. In HF with preserved ejection fraction (HFpEF), pre-specified analyses suggest that SGLT2 may similarly improve outcomes in patients with AF [76,77,78,79].

AF may lead to reduced cerebral blood flow, damaging the blood–brain barrier and promoting amyloid beta accumulation and tau protein dysfunction. These changes can accelerate neurodegeneration [80]. Studies using magnetic resonance imaging (MRI) have identified cerebral hypoperfusion in AF patients, with persistent AF showing more significant reductions in brain volume than paroxysmal AF. In a multicenter study of patients over 75 years with AF, brain volume reductions correlated with cerebral hypoperfusion, independent of traditional risk factors [81].

### 8.3. Complexities in Treatment Decisions

The EAST-AFNET4 study [36] highlights the complexities of rhythm control strategies in elderly patients with AF and HF. In this study, only 28% of participants had HF, and 80% of those in the rhythm control group received AADs, which carry a higher risk of adverse effects in elderly patients, including significant drug–drug interactions. Given the pathophysiological, prognostic, and prevalence overlaps of AF and HF in the elderly, treatment strategies must balance symptom management with the potential risks of therapy. Future studies should investigate outcomes beyond mortality, including quality of life, to better tailor interventions to the clinical phenotypes of elderly patients.

AF and HF are two characteristics of ageing pathologies in terms of their pathophysiology, prevalence, and prognostic impact on the elderly population. In patients with HF, the presence of a secondary diagnosis of AF is associated with a higher burden of symptoms, hospitalization, and mortality [82].

In this population, the choice between rhythm and rate control depends on the patient’s symptoms and each treatment option’s potential benefits and adverse events [82]. Further studies that include mortality and secondary endpoints, such as quality of life (QoL) according to the patient’s clinical phenotype, are desirable to gain a more complete understanding of the effects of the treatment under investigation in the elderly population [82].

## 9. AF and Dementia

### 9.1. Association Between AF and Cognitive Decline

Recent research has suggested a link between AF and dementia: the risk of developing dementia doubles in the presence of AF [83]. Several mechanisms have been implicated in the association between AF and dementia, including cerebral hypoperfusion, a pro-inflammatory state, endothelial dysfunction, microbleeds, and the occurrence of silent microinfarcts, which may contribute to progressive cognitive impairment. In elderly patients, the importance of the careful management of AF is evident in preventing thromboembolic events and reducing the risk of cognitive decline [84].

### 9.2. Pathophysiological Mechanisms

AF can contribute to reduced cerebral blood flow, which can lead to damage to the blood–brain barrier; it increases the metabolism of the amyloid beta precursor protein by reducing clearance and inducing senile plaques. It can also lead to local acidosis in the brain and increase oxidative stress, both of which can affect the function of the tau protein, leading to hyperphosphorylation and the formation of tau oligomers, factors which can accelerate cognitive impairment [85].

### 9.3. Clinical and Imaging Evidence

A comparative study using magnetic resonance imaging (MRI) found cerebral hypoperfusion, suggesting a possible link between cognitive impairment in the elderly and AF, and showed that cerebral perfusion was lower in persistent AF than in paroxysmal AF, indicating a reduction in brain volume [86]. The irregularity of the heartbeat could explain cerebral hypoperfusion and could be a cause of cognitive impairment [87].

In a study on elderly patients (>75 years) with AF, there was a reduction in brain volumes after correction for risk factors, which might be explained by cerebral hypoperfusion [88]. In the observational SAGE-AF study [53], 972 patients were assigned to either rhythm control or rate control. Those in the rate control group tended to be older, had a higher likelihood of persistent AF, a history of stroke, were more frequently on warfarin, and had cognitive impairment at the start of the study. After two years, participants receiving rate control were 1.5 times more likely to experience cognitive impairment compared to those treated with rhythm control [89].

### 9.4. Therapeutic Implications

Rhythm irregularity in AF may be the cause of cerebral hypoperfusion; therefore, to prevent dementia in selected elderly patients receiving optimal anticoagulant therapy, rhythm control may be preferable to rate control, taking into account clinical features and patient preference [86].

## 10. Anticoagulant Therapy in Elderly Patients

### 10.1. Thromboembolic Risk and the Role of Age

Age plays a critical role in determining the thromboembolic risk of elderly patients with AF, and as an independent factor, it alone justifies the use of oral anticoagulation therapy in these individuals [90,91]. Elderly patients are also more likely to present with multiple comorbidities, which further elevate their risk of stroke and systemic thromboembolism [90].

### 10.2. Evaluating Bleeding Risk

Regarding bleeding risk, older individuals tend to exhibit a higher burden of both modifiable and non-modifiable risk factors, such as anemia, frailty, renal dysfunction, and fall risk. The latest guidelines from the European Society of Cardiology (ESC) [6] emphasize the importance of evaluating bleeding risk in elderly patients but clarify that this should not dictate the decision to initiate or discontinue anticoagulant therapy. Rather, these assessments should guide the management of underlying conditions that contribute to bleeding risk [90]. Moreover, the guidelines do not advocate for the use of specific bleeding risk scores, as inconsistent results from other studies have limited their predictive value [92].

### 10.3. Barriers to Optimal Anticoagulation

AF is frequently diagnosed for the first time during hospitalization. Despite this, suboptimal prescribing practices persist due to factors including therapeutic inertia and biases among healthcare providers, particularly when treating elderly patients [93,94]. While adherence improved, the utilization of DOACs remains relatively low, particularly in patients with geriatric syndromes or high bleeding risk [95]. Another analysis highlighted that factors such as anemia, renal dysfunction, liver disease, antiarrhythmic drug use, and a high HAS-BLED score were associated with a lower likelihood of anticoagulant prescription [96].

### 10.4. Advantages of DOACs over Warfarin

The introduction of DOACs has undoubtedly facilitated increased anticoagulation use in this population (Table 2). Despite the limited representation of individuals aged ≥75 years in clinical trials, major international guidelines now recommend DOACs as a first-line therapy for stroke [97] in patients with AF, regardless of age [6,35]. A study by the Italian Society of Cardio-Geriatrics assessed the impact of anticoagulant therapy (warfarin or DOACs) in a large cohort of elderly patients (mean age 78.7 years) hospitalized for AF [98]. The analysis revealed that a significant proportion of these patients either received no antithrombotic treatment (24.8%) or were discharged on aspirin therapy (14.2%), both of which were associated with higher rates of ischemic stroke, systemic embolism, and mortality. Compared to warfarin, DOACs were associated with a lower incidence of cerebral hemorrhage and mortality, with no significant differences in cardioembolic events [99,100]. The study thus supports the net clinical benefit of [101] Cs over warfarin in elderly patients and underscores the need for educational campaigns to address the high rates of undertreatment or suboptimal therapy in this population [98].

In comparison with warfarin, DOACs offer a predictable pharmacokinetic profile, do not require regular monitoring, and have fewer drug–drug and drug–food interactions. However, the presence of end-stage renal disease or dialysis may limit the use of DOACs, as the optimal risk–benefit profile in these scenarios remains uncertain. Comorbidities and patient preferences must also be carefully considered when making anticoagulation decisions for elderly individuals [102].

### 10.5. Considerations in Frail Elderly Patients

While frailty itself is not a contraindication to anticoagulation therapy, the prescription of DOACs in elderly patients presents a challenge, as it is essential to assess their overall health status, cognitive function, and life expectancy [101]. Apixaban is generally considered the safest option, while dabigatran and rivaroxaban, according to the Beers Criteria, are associated with higher bleeding risk [103].

Factors contributing to the complexity of anticoagulation in frail elderly patients include anemia, prior bleeding events, advanced age, comorbidities, and the concurrent use of anti-inflammatory drugs [104]. A recent cohort study in frail AF patients found that the thromboembolic risk was similar between warfarin and DOACs. However, the risk of major bleeding was approximately 2% lower in patients treated with DOACs [105]. In this population, apixaban has been shown to reduce adverse clinical outcomes (ischemic stroke, systemic embolism, major hemorrhage, or death) and shorten hospitalization times compared to rivaroxaban and warfarin [106].

### 10.6. Warfarin in the Elderly

For elderly patients in whom DOACs are contraindicated and who must rely on vitamin K antagonists (VKAs), such as warfarin, it is essential to remember that their sensitivity to these drugs increases with age [101]. After the age of 70 years, the required dose of warfarin to maintain a therapeutic INR is reduced by approximately 20%, particularly in women [107,108,109,110]. Therefore, anticoagulant therapy in the elderly should be closely monitored, with regular re-evaluations of renal function and bleeding risk to prevent non-adherence or inappropriate dosing, as well as to minimize the risk of major bleeding (Table 3).

In conclusion, the decision to prescribe anticoagulation therapy in frail elderly patients with AF should be based on an individualized risk assessment, incorporating both thromboembolic and bleeding risk scores (such as CHA2DS2-VASc and HAS-BLED) alongside functional and cognitive evaluations. In patients at high risk of falls, anticoagulation decisions should prioritize these individual factors while taking into account the overall prognosis and treatment goals [115].

## 11. Decline in Quality of Life and Functional Impairment

The chronic symptoms of AF, such as palpitations, fatigue, and exercise intolerance, can markedly reduce quality of life (QoL) [116,117]. In elderly individuals, these symptoms may contribute to progressive functional decline and loss of independence, ultimately impacting both physical and psychological well-being [116,118].

## 12. Increased Hospitalizations and Healthcare Burden

The intermittent and unpredictable nature of AF often results in frequent hospital admissions—both for acute arrhythmia management and for addressing its complications [119]. This pattern increases healthcare costs and places a substantial burden on healthcare systems and caregivers [119].

## 13. Psychological Impact

AF can lead to significant psychological distress.

The persistent anxiety over recurrent episodes, combined with the fear of stroke or other complications, may predispose elderly patients to depression, social isolation, and overall diminished mental health.

## 14. Best-Practice Recommendations for Clinicians Managing Elderly AF Patients

Based on the evidence discussed in this manuscript, the below best-practice recommendations are proposed to guide clinicians in managing AF in the elderly. These recommendations emphasize the importance of a patient-centred individualized approach that accounts for the heterogeneity of this population.

### 14.1. Comprehensive Geriatric Assessment

Prior to initiating treatment, perform a thorough evaluation that includes assessments of frailty, cognitive function, nutritional status, and overall functional capacity.

Utilize standardized tools (e.g., FRAIL scale, Mini-Cog, MNA) to identify patients who are likely to benefit from tailored interventions while minimizing the risk of complications [120].

### 14.2. Individualized Rhythm vs. Rate Control Strategies

For patients with significant frailty, multiple comorbidities, or limited life expectancy, rate control may be favoured to reduce the risk of adverse events associated with antiarrhythmic drugs. In elderly individuals without frailty, particularly those with recent-onset AF and preserved functional status, rhythm control (whether pharmacological or via catheter-based interventions) may offer a reduction in adverse cardiovascular outcomes [121,122].

### 14.3. Cognitively Impaired Patients

Simplify treatment regimens and ensure close monitoring to enhance medication adherence and minimize the risk of errors, potentially favouring strategies that require less complex management [120].

### 14.4. Anticoagulation Therapy

Assess both thromboembolic (e.g., using CHA_2_DS_2_-VASc) and bleeding risks (e.g., using HAS-BLED) while incorporating clinical judgement regarding the patient’s overall health status, fall risk, and cognitive function [120].

Prefer DOACs over VKAs when possible due to their predictable pharmacokinetics and lower interaction potential. However, in patients with contraindications to DOACs (such as significant renal impairment), carefully adjusted warfarin therapy with rigorous monitoring remains a viable alternative [120].

### 14.5. Patient-Centred Decision-Making and Education

Engage patients and their caregivers in shared decision-making to align treatment goals with patient preferences and expectations, particularly in the context of cognitive impairment or frailty [120].

Clearly communicate the risks and benefits of each treatment option, emphasizing the balance between improving QoL and managing potential adverse events.

### 14.6. Regular Monitoring and Follow-Up

Schedule frequent follow-up visits to reassess clinical status, review medication adherence, and monitor for complications [110,123].

Adapt treatment strategies as patient conditions evolve, ensuring that adjustments in therapy are made in a timely manner to maintain optimal control of AF and its associated risks.

**Figure 4:**
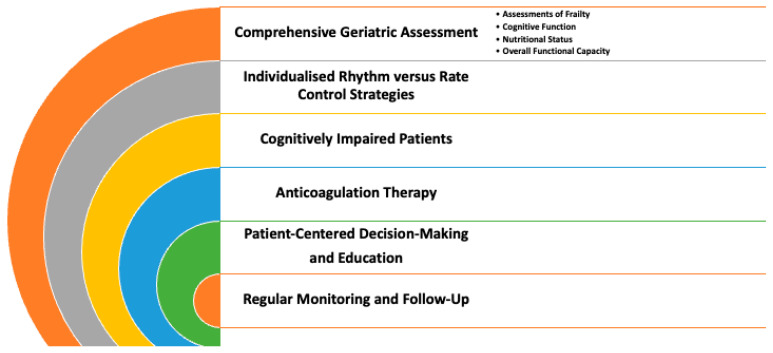
Practical framework for optimizing the management of AF in elderly patients.

These recommendations are designed to offer a practical framework for optimizing the management of AF in elderly patients, ensuring that therapeutic strategies are tailored to individual patient profiles and clinical contexts (Figure 4).

## 15. Age-Related Differences in AF Management

While the manuscript provides an in-depth discussion of AF in elderly patients, it offers limited direct comparisons with younger individuals despite significant differences between these populations. Older patients typically present with a higher burden of comorbidities, including obstructive sleep apnea [124], coronary artery disease [125], arterial hypertension [21], diabetes [126], and chronic kidney disease [126], which increase both thromboembolic and bleeding risks [120]. They also exhibit altered pharmacokinetics and a greater susceptibility to adverse drug effects, necessitating careful anticoagulation management. In contrast, younger AF patients often have fewer comorbidities and may tolerate rhythm control strategies more effectively. Given the increased risk of stroke in older adults, anticoagulation remains a cornerstone of treatment, with DOACs preferred over VKAs due to their superior safety profile. Additionally, rate control is often favoured in the elderly, as rhythm control strategies may be less effective and carry a higher risk of adverse events. These distinctions underscore the need for an individualized approach to AF management, optimizing both safety and efficacy in elderly patients.

## 16. Gaps in Evidence and Future Directions

Despite significant advancements in the management of AF in elderly patients, several critical gaps remain. Current randomized controlled trials often exclude or underrepresent patients over the age of 85 and those with multiple comorbidities, limiting the applicability of existing evidence to real-world clinical practice [5,6]. Moreover, the scarcity of data on the long-term outcomes of rate control versus rhythm control strategies in frail elderly populations makes it challenging to tailor treatment approaches for this vulnerable group. Anticoagulation management remains particularly complex, as current guidelines do not fully integrate frailty assessments or individualized bleeding risk stratification [5,6]. In this context, an area of great potential lies in the use of emerging technologies, including wearable devices and artificial intelligence (AI)-driven algorithms, which could play a crucial role in improving AF detection, risk stratification, and treatment monitoring in older adults [127,128,129]. AI, in particular, could contribute to an earlier and more accurate identification of patients at risk of AF episodes, enabling more targeted and personalized preventive interventions [127,128,129]. Furthermore, AI could optimize anticoagulation management, adjusting treatments in real time to meet each patient’s specific needs, taking into account frailty and individual risk factors [127,128,129].

Future research directions should also focus on refining multidisciplinary patient-centred care models, with the goal of optimizing clinical outcomes and QoL [3]. The integration of AI, wearable technologies, and personalized therapeutic approaches could revolutionize the management of AF in the elderly, enhancing not only treatment efficacy but also its adaptability to the specific needs of each individual. Addressing these gaps is crucial for developing more effective evidence-based strategies for AF management in the ageing population.

## 17. Conclusions

Although AF is common among the elderly, there is a lack of substantial evidence to inform clinical decisions for individuals aged 75 and older, with very little high-quality data available for those over 80 who have multiple co-existing conditions, physical or cognitive impairments, frailty, or who live in long-term care facilities.

Rate versus rhythm control for the optimal treatment of AF remains a hotly debated topic. Both have their merits and flaws, and clinical studies support their use.

Factors indicating that the patient would benefit most from rhythm control include age, significant AF symptoms, and HF. Rhythm control may be more appropriate in patients with multiple comorbidities, a low probability of long-term SR maintenance, and an enlarged left atrium.

Decisions must be made case-by-case after an informed decision-making process with the patient. The focus is frequently placed on pharmacological treatments, surgical options, and catheter-based procedures, while non-pharmacological approaches, such as diet, lifestyle changes, and exercise, receive far less attention.

The elderly population is highly heterogeneous, embracing independent individuals, disabled, and frail subjects.

In fit patients or those with non-severe frailty, rhythm control in the early phase of AF onset may be an effective strategy; however, the adverse effects of medications or complications related to invasive procedures as well as any conditions predisposing to adverse effects should be considered. Patient-centred decision-making has the most significant benefit, avoiding both futility and harm.

## Figures and Tables

**Table 1:** Summary of studies addressing AF in the elderly.

Author	Median Age (Years)	Number of Patients	Rate Control Strategy	Rhythm Control Strategy	Follow-Up Duration	Key Findings
Kirchoff [9]	63.7 (±10.9)	242	Without flecainide; persistent AF undergoing planned cardioversion	Flecainide	Short-term	Long-term antiarrhythmic drug treatment is more effective in preventing AF recurrence than short-term treatment.
Roy [10]	Not specified	1376	694 patients in rate control group	682 patients in rhythm control group	37 months	Rhythm control does not reduce cardiovascular mortality compared to rate control.
Van Gelder [11] (RACE)	68 (±9)	522	Rate control	Rhythm control	1 year	Rate control is not inferior to rhythm control for preventing cardiovascular death and morbidity.
Wyse [12] (AFFIRM)	70	4060	Rate control	Rhythm control	3.5 years	Rhythm control provides no survival advantage over rate control; higher risk of adverse drug effects.
Opolsky [13]	60.8 (±11.2)	205	Hospital admissions lower in rate control arm	No significant differences in major endpoints	1.7 years	There was no significant difference in major endpoints between the rate and rhythm control groups.
Tsadok [14]	75	16,325	Rate control	Rhythm control	2.8 years	Rhythm control is associated with lower stroke/TIA rates, particularly in high-risk patients.
Ionescu-Ittu [15]	66	26,130	Rate control	Rhythm control	2.3 years	Minimal mortality difference within 4 years; rhythm control superior in the long term.
Van Gender [16]	Not specified	698	614 patients in rate-control group	Not specified	3 years	Lenient rate control is both effective and strict and easier to achieve.

This table summarizes the key clinical trials comparing rate control and rhythm control strategies in elderly patients with AF. The evidence suggests that rate control is often non-inferior to rhythm control regarding mortality and cardiovascular outcomes. However, rhythm control may be beneficial in specific subgroups, such as those with a higher risk of stroke.

**Table 2:** Physiological age-related changes and their impact on DOACs in elderly patients with AF.

Physiological Change	Impact on DOACs and Prognosis
↓ Muscle mass and total body water (10–15%)	↑ Plasma concentration of hydrophilic DOACs (e.g., apixaban, edoxaban) → ⚠️ ↑ anticoagulant effect.
↓ Renal function (↓GFR)	↑ Plasma concentrations of all DOACs (greatest impact on dabigatran, predominantly renally excreted) → ⚠️ requires dose adjustment to prevent accumulation and bleeding risk.
↓ Hepatic function ↓ Liver size −25–35% ↓ Hepatic blood flow −40%	🔄 Altered drug metabolism → ⚠️ ↑ half-Life ↑ bleeding risk.
↑ Comorbidities ↑ bleeding risk ↑ frailty	⚠️ ↑ Bleeding risk even at therapeutic anticoagulant doses → requires individualized risk–benefit assessment.
↑ Thromboembolic risk	↑ Risk of ischemic stroke and systemic embolism. DOACs ✅ predictable pharmacokinetics ↓ intracranial bleeding risk, no need for routine monitoring.
↑ Polypharmacy and drug–drug interactions	🔄 ↑ Risk of pharmacokinetic interactions (e.g., P-glycoprotein and CYP3A4 inhibitors/inducers) → ⚠️ adverse effects.

↑ = increased; ↓ = decreased; ⚠️ = caution; 🔄 = variable effects; ✅ = preferred. This table highlights the complexities of DOAC therapy in elderly patients with AF, emphasizing the need for personalized anticoagulation strategies to balance thromboembolic protection and bleeding risk. Abbreviations: GFR: glomerular filtration rate; DOACs: direct oral anticoagulants.

**Table 3:** DOAC standard doses, adjustments, and clinical evidence.

DOAC	💊 Standard Dose	⚠️ Dose Adjustments	Evidence
Apixaban	5 mg twice daily	- 2.5 mg twice daily if ≥2 of the following: ▶️ Age ≥ 80 years ▶️ Body weight ≤ 60 kg ▶️ Serum creatinine ≥ 1.5 mg/dL (133 µmol/L) - Consider dose reduction if CrCl < 30 mL/min	ARISTOTLE trial [111] ✅ ↑ stroke prevention, ↓ major bleeding risk vs. warfarin ⚠️ Underdosing increases thromboembolic risk
Dabigatran	150 mg twice daily	- 110 mg twice daily in ▶️ Patients ≥ 80 years ▶️ High bleeding risk - Use 75 mg twice daily for CrCl 15–30 mL/min 🚫 Contraindicated if CrCl < 15 mL/min	RE-LY trial [112] ✅ Reduced stroke risk vs. warfarin ⚠️ ↑ gastrointestinal bleeding risk ⚠️ 80% renal clearance → dose adjustment critical
Edoxaban	60 mg once daily	- Reduce to 30 mg once daily if ▶️ CrCl 15–50 mL/min ▶️ Body weight ≤ 60 kg ▶️ Concomitant P-gp inhibitors 🚫 Avoid if CrCl > 95 mL/min (reduced efficacy) 🚫 Contraindicated if CrCl < 15 mL/min	ENGAGE AF-TIMI 48 trial [113] ✅ Non-inferior stroke prevention vs. warfarin ✅ ↓ major bleeding risk ⚠️ ↓ efficacy if CrCl > 95 mL/min → consider alternatives
Rivaroxaban	20 mg once daily	- 15 mg once daily if CrCl 15–49 mL/min 🚫 Contraindicated if CrCl < 15 mL/min - Consider dose adjustment in frail elderly with multiple risk factors	ROCKET-AF trial [114] ✅ Comparable stroke prevention vs. warfarin ⚠️ ↑ gastrointestinal bleeding risk ✅ Once-daily dosing improves adherence

Legend: 💊 = standard dose; ⚠️ = dose adjustments; 🚫 = contraindicated; ✅ = benefit; ↑ = increased; ↓ = reduced; CrCl: creatinine clearance. This table integrates critical evidence from pivotal clinical trials, ensuring an evidence-based approach to anticoagulation in elderly AF patients. Regularly assessing renal function (e.g., CrCl or eGFR) is crucial for dose optimization, especially in elderly patients with declining renal function. Moreover, in the elderly, a dose reduction may be necessary to balance bleeding and stroke risk. Underdosing increases ischemic stroke risk, whereas overdosing elevates bleeding complications—personalized anticoagulation strategies are recommended.

## References

[1] Fumagalli S., Potpara T.S., Bjerregaard Larsen T., Haugaa K.H., Dobreanu D., Proclemer A., Dagres N. (2017). Frailty syndrome: An emerging clinical problem in the everyday management of clinical arrhythmias. The results of the European Heart Rhythm Association survey. Europace.

[2] Bilato C., Corti M.C., Baggio G., Rampazzo D., Cutolo A., Iliceto S., Crepaldi G. (2009). Prevalence, functional impact, and mortality of atrial fibrillation in an older Italian population (from the Pro. V.A. study). Am. J. Cardiol..

[3] Lucà F., Abrignani M.G., Oliva F., Canale M.L., Parrini I., Murrone A., Rao C.M., Nesti M., Cornara S., Di Matteo I. (2024). Multidisciplinary Approach in Atrial Fibrillation: As Good as Gold. J. Clin. Med..

[4] Bodagh N., Kotadia I., Gharaviri A., Zelaya F., Birns J., Bhalla A., Sommerville P., Niederer S., O’Neill M., Williams S.E. (2023). The Impact of Atrial Fibrillation Treatment Strategies on Cognitive Function. J. Clin. Med..

[5] Joglar J.A., Chung M.K., Armbruster A.L., Benjamin E.J., Chyou J.Y., Cronin E.M., Deswal A., Eckhardt L.L., Goldberger Z.D., Gopinathannair R. (2024). 2023 ACC/AHA/ACCP/HRS Guideline for the Diagnosis and Management of Atrial Fibrillation: A Report of the American College of Cardiology/American Heart Association Joint Committee on Clinical Practice Guidelines. Circulation.

[6] Van Gelder I.C., Rienstra M., Bunting K.V., Casado-Arroyo R., Caso V., Crijns H., De Potter T.J.R., Dwight J., Guasti L., Hanke T. (2024). 2024 ESC Guidelines for the management of atrial fibrillation developed in collaboration with the European Association for Cardio-Thoracic Surgery (EACTS): Developed by the task force for the management of atrial fibrillation of the European Society of Cardiology (ESC), with the special contribution of the European Heart Rhythm Association (EHRA) of the ESC. Endorsed by the European Stroke Organisation (ESO). Eur. Heart J..

[7] Shamseer L., Moher D., Clarke M., Ghersi D., Liberati A., Petticrew M., Shekelle P., Stewart L.A. (2015). Preferred reporting items for systematic review and meta-analysis protocols (PRISMA-P) 2015: Elaboration and explanation. BMJ.

[8] Hutton B., Salanti G., Caldwell D.M., Chaimani A., Schmid C.H., Cameron C., Ioannidis J.P., Straus S., Thorlund K., Jansen J.P. (2015). The PRISMA extension statement for reporting of systematic reviews incorporating network meta-analyses of health care interventions: Checklist and explanations. Ann. Intern. Med..

[9] Kirchhof P., Andresen D., Bosch R., Borggrefe M., Meinertz T., Parade U., Ravens U., Samol A., Steinbeck G., Treszl A. (2012). Short-term versus long-term antiarrhythmic drug treatment after cardioversion of atrial fibrillation (Flec-SL): A prospective, randomised, open-label, blinded endpoint assessment trial. Lancet.

[10] Roy D., Talajic M., Nattel S., Wyse D.G., Dorian P., Lee K.L., Bourassa M.G., Arnold J.M., Buxton A.E., Camm A.J. (2008). Rhythm control versus rate control for atrial fibrillation and heart failure. N. Engl. J. Med..

[11] Van Gelder I.C., Groenveld H.F., Crijns H.J., Tuininga Y.S., Tijssen J.G., Alings A.M., Hillege H.L., Bergsma-Kadijk J.A., Cornel J.H., Kamp O. (2010). Lenient versus strict rate control in patients with atrial fibrillation. N. Engl. J. Med..

[12] Wyse D.G., Waldo A.L., DiMarco J.P., Domanski M.J., Rosenberg Y., Schron E.B., Kellen J.C., Greene H.L., Mickel M.C., Dalquist J.E. (2002). A comparison of rate control and rhythm control in patients with atrial fibrillation. N. Engl. J. Med..

[13] Opolski G., Torbicki A., Kosior D.A., Szulc M., Wozakowska-Kaplon B., Kolodziej P., Achremczyk P. (2004). Rate control vs. rhythm control in patients with nonvalvular persistent atrial fibrillation: The results of the Polish How to Treat Chronic Atrial Fibrillation (HOT CAFE) Study. Chest.

[14] Tsadok M.A., Jackevicius C.A., Essebag V., Eisenberg M.J., Rahme E., Humphries K.H., Tu J.V., Behlouli H., Pilote L. (2012). Rhythm versus rate control therapy and subsequent stroke or transient ischemic attack in patients with atrial fibrillation. Circulation.

[15] Ionescu-Ittu R., Abrahamowicz M., Jackevicius C.A., Essebag V., Eisenberg M.J., Wynant W., Richard H., Pilote L. (2012). Comparative effectiveness of rhythm control vs. rate control drug treatment effect on mortality in patients with atrial fibrillation. Arch. Intern. Med..

[16] Van Gelder I.C., Hagens V.E., Bosker H.A., Kingma J.H., Kamp O., Kingma T., Said S.A., Darmanata J.I., Timmermans A.J., Tijssen J.G. (2002). A comparison of rate control and rhythm control in patients with recurrent persistent atrial fibrillation. N. Engl. J. Med..

[17] Kistler P.M., Sanders P., Fynn S.P., Stevenson I.H., Spence S.J., Vohra J.K., Sparks P.B., Kalman J.M. (2004). Electrophysiologic and electroanatomic changes in the human atrium associated with age. JACC.

[18] Casaclang-Verzosa G., Gersh B.J., Tsang T.S.M. (2008). Structural and Functional Remodeling of the Left Atrium. JACC.

[19] Yamaguchi T. (2024). Atrial structural remodeling and atrial fibrillation substrate: A histopathological perspective. J. Cardiol..

[20] Gao P., Gao X., Xie B., Tse G., Liu T. (2024). Aging and atrial fibrillation: A vicious circle. Int. J. Cardiol..

[21] Soliman E.Z., Rahman A.F., Zhang Z.-M., Rodriguez C.J., Chang T.I., Bates J.T., Ghazi L., Blackshear J.L., Chonchol M., Fine L.J. (2020). Effect of Intensive Blood Pressure Lowering on the Risk of Atrial Fibrillation. Hypertension.

[22] Polidori M.C., Alves M., Bahat G., Boureau A.S., Ozkok S., Pfister R., Pilotto A., Veronese N., Bo M. (2022). Atrial fibrillation: A geriatric perspective on the 2020 ESC guidelines. Eur. Geriatr. Med..

[23] Aïdoud A., Gana W., Poitau F., Debacq C., Leroy V., Nkodo J.A., Poupin P., Angoulvant D., Fougère B. (2023). High Prevalence of Geriatric Conditions Among Older Adults with Cardiovascular Disease. J. Am. Heart Assoc..

[24] Dalgaard F., Xu H., Matsouaka R.A., Russo A.M., Curtis A.B., Rasmussen P.V., Ruwald M.H., Fonarow G.C., Lowenstern A., Hansen M.L. (2020). Management of Atrial Fibrillation in Older Patients by Morbidity Burden: Insights from Get with the Guidelines-Atrial Fibrillation. J. Am. Heart Assoc..

[25] Rockwood K., Song X., MacKnight C., Bergman H., Hogan D.B., McDowell I., Mitnitski A. (2005). A global clinical measure of fitness and frailty in elderly people. Cmaj.

[26] Thompson M.Q., Theou O., Tucker G.R., Adams R.J., Visvanathan R. (2020). FRAIL scale: Predictive validity and diagnostic test accuracy. Australas. J. Ageing.

[27] Moghtadaei M., Jansen H.J., Mackasey M., Rafferty S.A., Bogachev O., Sapp J.L., Howlett S.E., Rose R.A. (2016). The impacts of age and frailty on heart rate and sinoatrial node function. J. Physiol..

[28] Guigoz Y., Vellas B., Garry P.J. (1996). Assessing the nutritional status of the elderly: The Mini Nutritional Assessment as part of the geriatric evaluation. Nutr. Rev..

[29] Stewart C., Stewart J., Stubbs S., Epling J.W. (2022). Mini-Cog, IQCODE, MoCA, and MMSE for the Prediction of Dementia in Primary Care. Am. Fam. Physician.

[30] Volgman A.S., Nair G., Lyubarova R., Merchant F.M., Mason P., Curtis A.B., Wenger N.K., Aggarwal N.T., Kirkpatrick J.N., Benjamin E.J. (2022). Management of Atrial Fibrillation in Patients 75 Years and Older: JACC State-of-the-Art Review. J. Am. Coll. Cardiol..

[31] Lucà F., Parrini I. (2025). The Elderly Patient with Atrial Fibrillation: Optimal Treatment Strategies. J. Clin. Med..

[32] O’Mahony D., O’Sullivan D., Byrne S., O’Connor M.N., Ryan C., Gallagher P. (2015). STOPP/START criteria for potentially inappropriate prescribing in older people: Version 2. Age Ageing.

[33] Lavan A.H., Gallagher P., Parsons C., O’Mahony D. (2017). STOPPFrail (Screening Tool of Older Persons Prescriptions in Frail adults with limited life expectancy): Consensus validation. Age Ageing.

[34] Lucà F., La Meir M., Rao C.M., Parise O., Vasquez L., Carella R., Lorusso R., Daniela B., Maessen J., Gensini G.F. (2011). Pharmacological management of atrial fibrillation: One, none, one hundred thousand. Cardiol. Res. Pract..

[35] January C.T., Wann L.S., Calkins H., Chen L.Y., Cigarroa J.E., Cleveland J.C., Ellinor P.T., Ezekowitz M.D., Field M.E., Furie K.L. (2019). AHA/ACC/HRS Focused Update of the 2014 AHA/ACC/HRS Guideline for the Management of Patients with Atrial Fibrillation: A Report of the American College of Cardiology/American Heart Association Task Force on Clinical Practice Guidelines and the Heart Rhythm Society. J. Am. Coll. Cardiol..

[36] Kirchhof P., Camm A.J., Goette A., Brandes A., Eckardt L., Elvan A., Fetsch T., van Gelder I.C., Haase D., Haegeli L.M. (2020). Early Rhythm-Control Therapy in Patients with Atrial Fibrillation. N. Engl. J. Med..

[37] Noheria A., Shrader P., Piccini J.P., Fonarow G.C., Kowey P.R., Mahaffey K.W., Naccarelli G., Noseworthy P.A., Reiffel J.A., Steinberg B.A. (2016). Rhythm Control Versus Rate Control and Clinical Outcomes in Patients with Atrial Fibrillation: Results from the ORBIT-AF Registry. JACC Clin. Electrophysiol..

[38] Lucà F., Giubilato S., Di Fusco S.A., Piccioni L., Rao C.M., Iorio A., Cipolletta L., D’Elia E., Gelsomino S., Rossini R. (2021). Anticoagulation in Atrial Fibrillation Cardioversion: What Is Crucial to Take into Account. J. Clin. Med..

[39] Kim D., Yang P.S., You S.C., Sung J.H., Jang E., Yu H.T., Kim T.H., Pak H.N., Lee M.H., Lip G.Y.H. (2021). Treatment timing and the effects of rhythm control strategy in patients with atrial fibrillation: Nationwide cohort study. BMJ.

[40] Yu G.I., Kim D., Sung J.H., Jang E., Yu H.T., Kim T.H., Pak H.N., Lee M.H., Lip G.Y.H., Yang P.S. (2022). Impact of frailty on early rhythm control outcomes in older adults with atrial fibrillation: A nationwide cohort study. Front. Cardiovasc. Med..

[41] Gelsomino S., La Meir M., Lucà F., Lorusso R., Crudeli E., Vasquez L., Gensini G.F., Maessen J. (2012). Treatment of lone atrial fibrillation: A look at the past, a view of the present and a glance at the future. Eur. J. Cardiothorac. Surg..

[42] Dalgaard F., Pallisgaard J.L., Numé A.K., Lindhardt T.B., Gislason G.H., Torp-Pedersen C., Ruwald M.H. (2019). Rate or Rhythm Control in Older Atrial Fibrillation Patients: Risk of Fall-Related Injuries and Syncope. J. Am. Geriatr. Soc..

[43] (2023). American Geriatrics Society 2023 updated AGS Beers Criteria^®^ for potentially inappropriate medication use in older adults. J. Am. Geriatr. Soc..

[44] Hohnloser S.H., Connolly S.J., Crijns H.J., Page R.L., Seiz W., Torp-Petersen C. (2008). Rationale and design of ATHENA: A placebo-controlled, double-blind, parallel arm Trial to assess the efficacy of dronedarone 400 mg bid for the prevention of cardiovascular Hospitalization or death from any cause in patiENts with Atrial fibrillation/atrial flutter. J. Cardiovasc. Electrophysiol..

[45] Boriani G., Blomström-Lundqvist C., Hohnloser S.H., Bergfeldt L., Botto G.L., Capucci A., Lozano I.F., Goette A., Israel C.W., Merino J.L. (2019). Safety and efficacy of dronedarone from clinical trials to real-world evidence: Implications for its use in atrial fibrillation. Europace.

[46] Echt D.S., Liebson P.R., Mitchell L.B., Peters R.W., Obias-Manno D., Barker A.H., Arensberg D., Baker A., Friedman L., Greene H.L. (1991). Mortality and morbidity in patients receiving encainide, flecainide, or placebo. The Cardiac Arrhythmia Suppression Trial. N. Engl. J. Med..

[47] Yagnala N., Moreland-Head L., Zieminski J.J., Mara K., Macielak S. (2024). Assessment of Dofetilide or Sotalol Tolerability in the Elderly. J. Cardiovasc. Pharmacol. Ther..

[48] Kim Y.G., Lee H.S., Kim H., Kim M., Jeong J.H., Choi Y.Y., Shim J., Choi J.I., Kim Y.H. (2024). Association of Antiarrhythmic Drug Therapy with Syncope and Pacemaker Implantation in Patients with Atrial Fibrillation. J. Am. Coll. Cardiol..

[49] Dan G.-A., Martinez-Rubio A., Agewall S., Boriani G., Borggrefe M., Gaita F., van Gelder I., Gorenek B., Kaski J.C., Kjeldsen K. (2018). Antiarrhythmic drugs–clinical use and clinical decision making: A consensus document from the European Heart Rhythm Association (EHRA) and European Society of Cardiology (ESC) Working Group on Cardiovascular Pharmacology, endorsed by the Heart Rhythm Society (HRS), Asia-Pacific Heart Rhythm Society (APHRS) and International Society of Cardiovascular Pharmacotherapy (ISCP). EP Eur..

[50] Hindricks G., Potpara T., Dagres N., Arbelo E., Bax J.J., Blomström-Lundqvist C., Boriani G., Castella M., Dan G.A., Dilaveris P.E. (2021). 2020 ESC Guidelines for the diagnosis and management of atrial fibrillation developed in collaboration with the European Association for Cardio-Thoracic Surgery (EACTS): The Task Force for the diagnosis and management of atrial fibrillation of the European Society of Cardiology (ESC) Developed with the special contribution of the European Heart Rhythm Association (EHRA) of the ESC. Eur. Heart J..

[51] Depoorter L., Sels L., Deschodt M., Van Grootven B., Van der Linden L., Tournoy J. (2020). Clinical Outcomes of Rate vs. Rhythm Control for Atrial Fibrillation in Older People: A Systematic Review and Meta-Analysis. Drugs Aging.

[52] Vermond R.A., Crijns H.J., Tijssen J.G., Alings A.M., Van den Berg M.P., Hillege H.L., Van Veldhuisen D.J., Van Gelder I.C., Rienstra M. (2014). Symptom severity is associated with cardiovascular outcome in patients with permanent atrial fibrillation in the RACE II study. Europace.

[53] Parrini I., Lucà F., Rao C.M., Cacciatore S., Riccio C., Grimaldi M., Gulizia M.M., Oliva F., Andreotti F. (2024). How to Manage Beta-Blockade in Older Heart Failure Patients: A Scoping Review. J. Clin. Med..

[54] Brophy J.M. (2006). Rehabilitating digoxin. Eur. Heart J..

[55] Bauman J.L., DiDomenico R.J., Viana M., Fitch M. (2006). A method of determining the dose of digoxin for heart failure in the modern era. Arch. Intern. Med..

[56] Marrouche N.F., Brachmann J., Andresen D., Siebels J., Boersma L., Jordaens L., Merkely B., Pokushalov E., Sanders P., Proff J. (2018). Catheter Ablation for Atrial Fibrillation with Heart Failure. N. Engl. J. Med..

[57] Boehmer A.A., Rothe M., Ruckes C., Eckardt L., Kaess B.M., Ehrlich J.R. (2024). Catheter Ablation for Atrial Fibrillation in Elderly Patients: An Updated Meta-analysis of Comparative Studies. Can. J. Cardiol..

[58] Sohns C., Marrouche N.F., Crijns H., Sossalla S., Sciacca V., Tijssen J.G.P., Sommer P. (2024). In CASTLE-HTx Trial We Trust. J. Am. Coll. Cardiol..

[59] Di Biase L., Mohanty P., Mohanty S., Santangeli P., Trivedi C., Lakkireddy D., Reddy M., Jais P., Themistoclakis S., Dello Russo A. (2016). Ablation Versus Amiodarone for Treatment of Persistent Atrial Fibrillation in Patients with Congestive Heart Failure and an Implanted Device: Results from the AATAC Multicenter Randomized Trial. Circulation.

[60] Mark J.D., Colombo R.A., Alfonso C.E., Llanos A., Collado E., Larned J.M., Giese G., Dyal M.D., Nanna M.G., Damluji A.A. (2024). The Impact of Frailty on Patients with AF and HFrEF Undergoing Catheter Ablation. JACC Adv..

[61] Nesti M., Lucà F., Duncker D., De Sensi F., Malaczynska-Rajpold K., Behar J.M., Waldmann V., Ammar A., Mirizzi G., Garcia R. (2023). Antiplatelet and Anti-Coagulation Therapy for Left-Sided Catheter Ablations: What Is beyond Atrial Fibrillation?. J. Clin. Med..

[62] Packer D.L., Mark D.B., Robb R.A., Monahan K.H., Bahnson T.D., Poole J.E., Noseworthy P.A., Rosenberg Y.D., Jeffries N., Mitchell L.B. (2019). Effect of Catheter Ablation vs. Antiarrhythmic Drug Therapy on Mortality, Stroke, Bleeding, and Cardiac Arrest Among Patients with Atrial Fibrillation: The CABANA Randomized Clinical Trial. JAMA.

[63] Palmisano P., Ziacchi M., Dell’Era G., Donateo P., Ammendola E., Aspromonte V., Pellegrino P.L., Del Giorno G., Coluccia G., Bartoli L. (2023). Ablate and pace: Comparison of outcomes between conduction system pacing and biventricular pacing. Pacing Clin. Electrophysiol..

[64] Joza J., Burri H., Andrade J.G., Linz D., Ellenbogen K.A., Vernooy K. (2024). Atrioventricular node ablation for atrial fibrillation in the era of conduction system pacing. Eur. Heart J..

[65] Nesti M., Luca F., Panchetti L., Garibaldi S., Startari U., Mirizzi G., Landra F., Giannoni A., Piacenti M., Rossi A. (2023). Impact of Vein of Marshall Ethanol Infusion Combined with Anatomical Ablation for the Treatment of Persistent Atrial Fibrillation: A Long-Term Follow-Up Based on Implantable Loop Recorders. J. Clin. Med..

[66] Miyazaki S. (2024). Catheter ablation of atrial fibrillation for frail patients. J. Cardiovasc. Electrophysiol..

[67] Ali S., Kumar M., Duhan S., Farooq F., Hendricks E., Keisham B., Manan M., Malik A., Talpur A.S., Latchana S. (2024). IMPACT OF FRAILTY ON SHORT-TERM OUTCOMES OF CATHETER ABLATION FOR ATRIAL FIBRILLATION: A PROPENSITY-MATCHED NATIONAL STUDY. JACC.

[68] Santangeli P., Biase L.D., Mohanty P., Burkhardt J.D., Horton R., Bai R., Mohanty S., Pump A., Gibson D., Couts L. (2012). Catheter Ablation of Atrial Fibrillation in Octogenarians: Safety and Outcomes. J. Cardiovasc. Electrophysiol..

[69] Xu R., Dong Y., Yadav N., Chen Q., Cao K., Zhang F. (2024). Prediction of Recurrence of Atrial Fibrillation After Radiofrequency Ablation by Frailty. J. Am. Heart Assoc..

[70] Karakasis P., Pamporis K., Siontis K.C., Theofilis P., Samaras A., Patoulias D., Stachteas P., Karagiannidis E., Stavropoulos G., Tzikas A. (2024). Major clinical outcomes in symptomatic vs. asymptomatic atrial fibrillation: A meta-analysis. Eur. Heart J..

[71] Odutayo A., Wong C.X., Hsiao A.J., Hopewell S., Altman D.G., Emdin C.A. (2016). Atrial fibrillation and risks of cardiovascular disease, renal disease, and death: Systematic review and meta-analysis. BMJ.

[72] Ruddox V., Sandven I., Munkhaugen J., Skattebu J., Edvardsen T., Otterstad J.E. (2017). Atrial fibrillation and the risk for myocardial infarction, all-cause mortality and heart failure: A systematic review and meta-analysis. Eur. J. Prev. Cardiol..

[73] Santhanakrishnan R., Wang N., Larson M.G., Magnani J.W., McManus D.D., Lubitz S.A., Ellinor P.T., Cheng S., Vasan R.S., Lee D.S. (2016). Atrial Fibrillation Begets Heart Failure and Vice Versa: Temporal Associations and Differences in Preserved Versus Reduced Ejection Fraction. Circulation.

[74] Coats A.J.S., Heymans S., Farmakis D., Anker S.D., Backs J., Bauersachs J., de Boer R.A., Čelutkienė J., Cleland J.G.F., Dobrev D. (2021). Atrial disease and heart failure: The common soil hypothesis proposed by the Heart Failure Association of the European Society of Cardiology. Eur. Heart J..

[75] Abrignani M.G., Aiello A., Colivicchi F., Lucà F., Fattirolli F., Gulizia M.M., Nardi F., Pino P.G., Gregorio G. (2020). Cardiovascular prevention in the elderly: Limitations and opportunities. G. Ital. Cardiol..

[76] Solomon S.D., McMurray J.J.V., Claggett B., de Boer R.A., DeMets D., Hernandez A.F., Inzucchi S.E., Kosiborod M.N., Lam C.S.P., Martinez F. (2022). Dapagliflozin in Heart Failure with Mildly Reduced or Preserved Ejection Fraction. N. Engl. J. Med..

[77] Anker S.D., Butler J., Filippatos G., Ferreira J.P., Bocchi E., Böhm M., Brunner-La Rocca H.P., Choi D.J., Chopra V., Chuquiure-Valenzuela E. (2021). Empagliflozin in Heart Failure with a Preserved Ejection Fraction. N. Engl. J. Med..

[78] Bhatt D.L., Szarek M., Steg P.G., Cannon C.P., Leiter L.A., McGuire D.K., Lewis J.B., Riddle M.C., Voors A.A., Metra M. (2021). Sotagliflozin in Patients with Diabetes and Recent Worsening Heart Failure. N. Engl. J. Med..

[79] Lucà F., Oliva F., Abrignani M.G., Di Fusco S.A., Gori M., Giubilato S., Ceravolo R., Temporelli P.L., Cornara S., Rao C.M. (2024). Heart Failure with Preserved Ejection Fraction: How to Deal with This Chameleon. J. Clin. Med..

[80] Tublin J.M., Adelstein J.M., del Monte F., Combs C.K., Wold L.E. (2019). Getting to the Heart of Alzheimer Disease. Circ. Res..

[81] Berman J.P., Norby F.L., Mosley T., Soliman E.Z., Gottesman R.F., Lutsey P.L., Alonso A., Chen L.Y. (2019). Atrial Fibrillation and Brain Magnetic Resonance Imaging Abnormalities. Stroke.

[82] Anter E., Jessup M., Callans D.J. (2009). Atrial Fibrillation and Heart Failure. Circulation.

[83] Ott A., Breteler M.M., de Bruyne M.C., van Harskamp F., Grobbee D.E., Hofman A. (1997). Atrial fibrillation and dementia in a population-based study. The Rotterdam Study. Stroke.

[84] Kalantarian S., Stern T.A., Mansour M., Ruskin J.N. (2013). Cognitive impairment associated with atrial fibrillation: A meta-analysis. Ann. Intern. Med..

[85] Carbone G., Ercolano E., Bencivenga L., Palaia M.E., Scognamiglio F., Rengo G., Femminella G.D. (2024). Atrial Fibrillation and Dementia: Focus on Shared Pathophysiological Mechanisms and Therapeutic Implications. J. Am. Med. Dir. Assoc..

[86] Rivard L., Friberg L., Conen D., Healey J.S., Berge T., Boriani G., Brandes A., Calkins H., Camm A.J., Yee Chen L. (2022). Atrial Fibrillation and Dementia: A Report from the AF-SCREEN International Collaboration. Circulation.

[87] Gardarsdottir M., Sigurdsson S., Aspelund T., Rokita H., Launer L.J., Gudnason V., Arnar D.O. (2018). Atrial fibrillation is associated with decreased total cerebral blood flow and brain perfusion. Europace.

[88] Moazzami K., Shao I.Y., Chen L.Y., Lutsey P.L., Jack C.R., Mosley T., Joyner D.A., Gottesman R., Alonso A. (2020). Atrial Fibrillation, Brain Volumes, and Subclinical Cerebrovascular Disease (from the Atherosclerosis Risk in Communities Neurocognitive Study [ARIC-NCS]). Am. J. Cardiol..

[89] Athreya D.S., Saczynski J.S., Gurwitz J.H., Monahan K.M., Bamgbade B.A., Paul T.J., Sogade F., Lessard D.M., McManus D.D., Helm R.H. (2024). Cognitive impairment and treatment strategy for atrial fibrillation in older adults: The SAGE-AF study. J. Am. Geriatr. Soc..

[90] Patti G., Lucerna M., Pecen L., Siller-Matula J.M., Cavallari I., Kirchhof P., De Caterina R. (2017). Thromboembolic Risk, Bleeding Outcomes and Effect of Different Antithrombotic Strategies in Very Elderly Patients with Atrial Fibrillation: A Sub-Analysis from the PREFER in AF (PRE vention o F Thromboembolic Events–E uropean R egistry in A trial F ibrillation). J. Am. Heart Assoc..

[91] Pozzi A., Lucà F., Gelsomino S., Abrignani M.G., Giubilato S., Di Fusco S.A., Rao C.M., Cornara S., Caretta G., Ceravolo R. (2024). Coagulation Tests and Reversal Agents in Patients Treated with Oral Anticoagulants: The Challenging Scenarios of Life-Threatening Bleeding and Unplanned Invasive Procedures. J. Clin. Med..

[92] Gao X., Cai X., Yang Y., Zhou Y., Zhu W. (2021). Diagnostic Accuracy of the HAS-BLED Bleeding Score in VKA- or DOAC-Treated Patients with Atrial Fibrillation: A Systematic Review and Meta-Analysis. Front. Cardiovasc. Med..

[93] Troy A.L., Herzig S.J., Trivedi S., Anderson T.S. (2023). Initiation of oral anticoagulation in US older adults newly diagnosed with atrial fibrillation during hospitalization. J. Am. Geriatr. Soc..

[94] Bhat A., Karthikeyan S., Chen H.H.L., Gan G.C.H., Denniss A.R., Tan T.C. (2023). Barriers to Guideline-Directed Anticoagulation in Patients with Atrial Fibrillation: New Approaches to an Old Problem. Can. J. Cardiol..

[95] Ko D., Lin K.J., Bessette L.G., Lee S.B., Walkey A.J., Cheng S., Kim E., Glynn R.J., Kim D.H. (2022). Trends in Use of Oral Anticoagulants in Older Adults with Newly Diagnosed Atrial Fibrillation, 2010-2020. JAMA Netw. Open.

[96] Manning E., Burns K., Laurie M., Patten L., Ho M., Sandhu A. (2023). Factors associated with oral anticoagulant prescription status among patients with a new diagnosis of atrial fibrillation. Clin. Cardiol..

[97] Abrignani M.G., Lucà F., Abrignani V., Pelaggi G., Aiello A., Colivicchi F., Fattirolli F., Gulizia M.M., Nardi F., Pino P.G. (2024). A Look at Primary and Secondary Prevention in the Elderly: The Two Sides of the Same Coin. J. Clin. Med..

[98] Bo M., Fumagalli S., Degli Esposti L., Perrone V., Dovizio M., Poli D., Marcucci R., Verdecchia P., Reboldi G., Lip G.Y.H. (2024). Anticoagulation in atrial fibrillation. A large real-world update. Eur. J. Intern. Med..

[99] Lucà F., Andreotti F., Rao C.M., Pelaggi G., Nucara M., Ammendolea C., Pezzi L., Ingianni N., Murrone A., Del Sindaco D. (2024). Acute Coronary Syndrome in Elderly Patients: How to Tackle Them?. J. Clin. Med..

[100] Lucà F., Colivicchi F., Oliva F., Abrignani M., Caretta G., Di Fusco S.A., Giubilato S., Cornara S., Di Nora C., Pozzi A. (2023). Management of oral anticoagulant therapy after intracranial hemorrhage in patients with atrial fibrillation. Front. Cardiovasc. Med..

[101] Botto G.L., Capranzano P., Colonna P., Fornasari D.M.M., Sciatti E., Riva L. (2024). Use of DOACs in frail elderly patients in light of class genericization. Int. J. Cardiol..

[102] Shields A.M., Lip G.Y. (2015). Choosing the right drug to fit the patient when selecting oral anticoagulation for stroke prevention in atrial fibrillation. J. Intern. Med..

[103] Mentias A., Heller E., Vaughan Sarrazin M. (2020). Comparative Effectiveness of Rivaroxaban, Apixaban, and Warfarin in Atrial Fibrillation Patients with Polypharmacy. Stroke.

[104] Stuby J., Haschke M., Tritschler T., Aujesky D. (2024). Oral anticoagulant therapy in older adults. Thromb. Res..

[105] Søgaard M., Ording A.G., Skjøth F., Larsen T.B., Nielsen P.B. (2024). Effectiveness and safety of direct oral anticoagulation vs. warfarin in frail patients with atrial fibrillation. Eur. Heart J. Cardiovasc. Pharmacother..

[106] Lin K.J., Singer D.E., Ko D., Glynn R., Najafzadeh M., Lee S.B., Bessette L.G., Cervone A., DiCesare E., Kim D.H. (2023). Frailty, Home Time, and Health Care Costs in Older Adults with Atrial Fibrillation Receiving Oral Anticoagulants. JAMA Netw. Open.

[107] Shendre A., Parmar G.M., Dillon C., Beasley T.M., Limdi N.A. (2018). Influence of Age on Warfarin Dose, Anticoagulation Control, and Risk of Hemorrhage. Pharmacotherapy.

[108] Lucà F., Abrignani M.G., Parrini I., Di Fusco S.A., Giubilato S., Rao C.M., Piccioni L., Cipolletta L., Passaretti B., Giallauria F. (2022). Update on Management of Cardiovascular Diseases in Women. J. Clin. Med..

[109] Lucà F., Pavan D., Gulizia M.M., Manes M.T., Abrignani M.G., Benedetto F.A., Bisceglia I., Brigido S., Caldarola P., Calvanese R. (2024). Italian Association of Hospital Cardiologists Position Paper ‘Gender discrepancy: Time to implement gender-based clinical management’. Eur. Heart J. Suppl..

[110] Lucà F., Pavan D., Gulizia M.M., Manes M.T., Abrignani M.G., Benedetto F.A., Bisceglia I., Brigido S., Caldarola P., Calvanese R. (2024). Position paper ANMCO: Differenze di genere nell’approccio farmacologico cardiovascolare. G. Ital. Di Cardiol..

[111] Granger C.B., Alexander J.H., McMurray J.J.V., Lopes R.D., Hylek E.M., Hanna M., Al-Khalidi H.R., Ansell J., Atar D., Avezum A. (2011). Apixaban versus Warfarin in Patients with Atrial Fibrillation. N. Engl. J. Med..

[112] Connolly S.J., Ezekowitz M.D., Yusuf S., Eikelboom J., Oldgren J., Parekh A., Pogue J., Reilly P.A., Themeles E., Varrone J. (2009). Dabigatran versus Warfarin in Patients with Atrial Fibrillation. N. Engl. J. Med..

[113] Giugliano R.P., Ruff C.T., Braunwald E., Murphy S.A., Wiviott S.D., Halperin J.L., Waldo A.L., Ezekowitz M.D., Weitz J.I., Špinar J. (2013). Edoxaban versus Warfarin in Patients with Atrial Fibrillation. N. Engl. J. Med..

[114] Patel M.R., Mahaffey K.W., Garg J., Pan G., Singer D.E., Hacke W., Breithardt G., Halperin J.L., Hankey G.J., Piccini J.P. (2011). Rivaroxaban versus Warfarin in Nonvalvular Atrial Fibrillation. N. Engl. J. Med..

[115] Guzzo A.S., Meggiolaro A., Mannocci A., Tecca M., Salomone I., La Torre G. (2015). Conley Scale: Assessment of a fall risk prevention tool in a General Hospital. J. Prev. Med. Hyg..

[116] Magnani J.W., Wang N., Benjamin E.J., Garcia M.E., Bauer D.C., Butler J., Ellinor P.T., Kritchevsky S., Marcus G.M., Newman A. (2016). Atrial Fibrillation and Declining Physical Performance in Older Adults: The Health, Aging, and Body Composition Study. Circ. Arrhythm. Electrophysiol..

[117] van Breugel H., Parise O., Nieman F.H.M., Accord R.E., Lucà F., Lozekoot P., Kumar N., van Mastrigt G., Nijs J., Vrakking R. (2016). Does sinus rhythm conversion after cardiac surgery affect postoperative health- related quality of life?. J. Cardiothorac. Surg..

[118] Tzeis S., Gerstenfeld E.P., Kalman J., Saad E.B., Sepehri Shamloo A., Andrade J.G., Barbhaiya C.R., Baykaner T., Boveda S., Calkins H. (2024). 2024 European Heart Rhythm Association/Heart Rhythm Society/Asia Pacific Heart Rhythm Society/Latin American Heart Rhythm Society expert consensus statement on catheter and surgical ablation of atrial fibrillation. EP Eur..

[119] Pastori D., Farcomeni A., Pignatelli P., Violi F., Lip G.Y.H. (2019). ABC (Atrial fibrillation Better Care) Pathway and Healthcare Costs in Atrial Fibrillation: The ATHERO-AF Study. Am. J. Med..

[120] Brunetti E., Lucà F., Presta R., Marchionni N., Boccanelli A., Ungar A., Rao C.M., Ingianni N., Lettino M., Del Sindaco D. (2024). A Comprehensive Geriatric Workup and Frailty Assessment in Older Patients with Severe Aortic Stenosis. J. Clin. Med..

[121] Gupta D., Rienstra M., van Gelder I.C., Fauchier L. (2024). Atrial fibrillation: Better symptom control with rate and rhythm management. Lancet Reg. Health–Eur..

[122] Klamer T.A., Bots S.H., Neefs J., Tulevski I.I., Ruijter H.M.d., Somsen G.A., de Groot J.R. (2022). Rate and Rhythm Control Treatment in the Elderly and Very Elderly Patients with Atrial Fibrillation: An Observational Cohort Study of 1497 Patients. Curr. Probl. Cardiol..

[123] Lucà F., Cipolletta L., Di Fusco S.A., Iorio A., Pozzi A., Rao C.M., Ingianni N., Benvenuto M., Madeo A., Fiscella D. (2019). Remote monitoring: Doomed to let down or an attractive promise?. Int. J. Cardiol. Heart Vasc..

[124] Moula A.I., Parrini I., Tetta C., Lucà F., Parise G., Rao C.M., Mauro E., Parise O., Matteucci F., Gulizia M.M. (2022). Obstructive Sleep Apnea and Atrial Fibrillation. J. Clin. Med..

[125] Lucà F., Caretta G., Vagnarelli F., Marini M., Iorio A., Di Fusco S.A., Pozzi A., Gabrielli D., Colivicchi F., De Luca L. (2020). Clinical characteristics, management and outcomes of patients with acute coronary syndrome and atrial fibrillation: Real-world data from two nationwide registries in Italy. J. Cardiovasc. Med..

[126] Seyed Ahmadi S., Svensson A.M., Pivodic A., Rosengren A., Lind M. (2020). Risk of atrial fibrillation in persons with type 2 diabetes and the excess risk in relation to glycaemic control and renal function: A Swedish cohort study. Cardiovasc. Diabetol..

[127] Spooner M.T., Messé S.R., Chaturvedi S., Do M.M., Gluckman T.J., Han J.K., Russo A.M., Saxonhouse S.J., Wiggins N.B. (2025). 2024 ACC Expert Consensus Decision Pathway on Practical Approaches for Arrhythmia Monitoring After Stroke. JACC.

[128] Mäkynen M., Ng G.A., Li X., Schlindwein F.S. (2022). Wearable Devices Combined with Artificial Intelligence—A Future Technology for Atrial Fibrillation Detection?. Sensors.

[129] Fiorina L., Chemaly P., Cellier J., Said M.A., Coquard C., Younsi S., Salerno F., Horvilleur J., Lacotte J., Manenti V. (2024). Artificial intelligence–based electrocardiogram analysis improves atrial arrhythmia detection from a smartwatch electrocardiogram. Eur. Heart J. Digit. Health.

